# Genomic characterization and genomic epidemiology of *Staphylococcus aureus* in chicken farms in Sichuan, China

**DOI:** 10.3389/fmicb.2026.1950719

**Published:** 2026-10-14

**Authors:** Yang Zhao, Yao Zhang, Jie Ma, Jie Zou, Manshan Cai, Yuting Li, Yuting Wang, Hao Qu, Yong Huang

**Affiliations:** 1 College of Veterinary Medicine, Sichuan Agricultural University, Chengdu, Sichuan, China; 2 Sichuan Dekon Agriculture and Animal Husbandry Food Group Co., Ltd., Chengdu, Sichuan, China; 3 Guangdong Wiz Agricultural Science & Technology Co. Ltd., Guangzhou, China; 4 Guangdong Academy of Agricultural Sciences, Guangzhou, China

**Keywords:** antimicrobial resistance, epidemiology, potential clonal transmission, *Staphylococcus aureus*, whole-genome sequencing

## Abstract

*Staphylococcus aureus (S. aureus)* is an important zoonotic opportunistic pathogen, and its transmission risks in poultry production systems have attracted increasing attention in recent years. In this study, 126 *S. aureus* isolates recovered from clinical and farm environmental samples were subjected to whole-genome sequencing and comparative genomic analysis to characterize their genotypes, antimicrobial resistance, virulence, and transmission patterns. The *spa* typing identified t2582 as the predominant type, whereas multi-locus sequence typing identified ST398 as the dominant sequence type. Overall, 35 antimicrobial resistance genes (ARGs) and 41 virulence-associated genes were detected. Only five isolates carried *mecA*, indicating the emergence of methicillin-resistant *S. aureus* (MRSA) isolates on chicken farms in Sichuan Province, China. Aminoglycoside- and tetracycline-resistance genes were significantly enriched in environmental isolates, whereas the number of virulence genes did not differ significantly between clinical and environmental isolates. Core-genome single-nucleotide polymorphism (SNP) analysis grouped the isolates into two major lineages, with clinical and environmental isolates interspersed throughout the phylogeny. Some clinical and environmental isolates showed extremely small pairwise SNP differences, suggesting potential transmission of *S. aureus* across difference sources. These findings demonstrate close epidemiological links between clinical and environmental isolates, highlighting the farm environment as a potential reservoir and transmission interface for *S. aureus*. Continuous genomic surveillance, improved environmental sanitation and enhanced biosecurity are therefore essential for controlling its spread in chicken farms.

## 1 Introduction

*Staphylococcus aureus* is an important Gram-positive bacterium that is widely distributed among humans, animals, and livestock production environments (Cheung et al., 2021). It can colonize the skin and mucosal surfaces as a commensal organism but may also cause infection when host barrier integrity is compromised, immune function is impaired, or environmental stress occurs (Horn and Kielian, 2020; Wang and Yang, 2024; Zhou et al., 2025). In poultry production, *S. aureus* and other staphylococcal species can cause both localized and systemic infections, with clinical manifestations including septicemia, arthritis, synovitis, osteomyelitis, omphalitis, gangrenous dermatitis, and pododermatitis (Bortolaia et al., 2016; Lowder et al., 2009; Qian et al., 2022). These diseases can result in substantial economic losses to the poultry industry. The bacterium can persist for prolonged periods on host body surfaces and in feces, dust, litter, drinking-water systems, and farm facilities (Shrestha et al., 2026; Wladyka et al., 2013). Consequently, the farm environment may serve not only as a reservoir for persistent bacterial contamination but also as an important vehicle for transmission among different flocks, production stages, and sample sources.

Antimicrobial resistance is one of the major challenges in the research, prevention, and control of *S. aureus*. The use of antimicrobial agents in livestock and poultry production can impose selective pressure on bacterial populations, thereby facilitating the dissemination of resistant strains and antimicrobial resistance genes among animals, farm environments, and occupationally exposed personnel (Panesso et al., 2015; Zhang et al., 2022). Both the World Health Organization and the World Organization for Animal Health have emphasized the importance of strengthening surveillance of antimicrobial resistance in animal-associated bacteria within the One Health framework to reduce the risk of resistant pathogens spreading among animals, humans, and the environment (Zinsstag et al., 2023). Methicillin-resistant *S. aureus* (MRSA) is of particular public health concern because it carries resistance determinants such as *mecA* (Carfora et al., 2016; Ledda et al., 2017; Patel et al., 2013). In addition, livestock-associated clonal lineages, including ST398, have been reported in pigs, cattle, poultry, and their associated environments and are therefore important targets for assessing the transmission risks of animal-associated *S. aureus* (Hu et al., 2025; Khairullah et al., 2024; Lee et al., 2024).

With advances in whole-genome sequencing (WGS), conventional *spa* typing and multilocus sequence typing (MLST) can be combined with core-genome SNP analysis to characterize the population structure and infer potential transmission chains of *S. aureus*, while sequence homology-based annotation against curated databases allows genotypic profiling of its ARGs and virulence gene repertoires (Landman et al., 2024). Compared with conventional typing methods, SNP distance-based genomic epidemiological analysis provides substantially higher resolution, enabling the identification of closely related isolates and the inference of potential recent transmission events (Gorrie et al., 2021). Previous studies have also indicated that SNP thresholds should be interpreted in conjunction with the pathogen species, epidemiological context, and spatiotemporal sampling information (Forde et al., 2023; Steinig et al., 2022). Nevertheless, the transmission relationships between clinical and environmental *S. aureus* isolates from chicken farms, differences in antimicrobial resistance gene distribution, and the dissemination patterns of predominant clonal lineages remain insufficiently characterized.

Therefore, in the present study, *S. aureus* isolates were recovered from clinical and environmental samples collected from chicken farms in selected regions, followed by WGS and comparative genomic analyses. Emphasis was placed on characterizing their molecular typing profiles, antimicrobial resistance and virulence gene repertoires, phylogenetic relationships, and potential transmission events. The findings of this study provide insight into the epidemiological patterns and cross-transmission risks of chicken-associated *S. aureus* within chicken farms and offer a scientific basis for antimicrobial resistance surveillance and the development of targeted prevention and control strategies in poultry production.

## 2 Materials and methods

### 2.1 Sample collection and isolation of *Staphylococcus aureus*

Between January 2025 and June 2026, a total of 1,444 samples were collected from commercial chicken farms in Sichuan Province, China. The samples comprised specimens from clinical cases and samples collected from the farm environment (Table 1). Samples were collected using sterile cotton swabs pre-moistened with sterile physiological saline. For joint specimens, the tarsal joint was aseptically incised using a sterile scalpel, after which the exposed joint cavity and surrounding tissues were swabbed with a sterile cotton applicator. All samples were immediately transported to the laboratory under refrigerated conditions at 4 °C, and bacterial isolation was performed within 48 h of collection. For bacterial isolation, each sample was initially inoculated into 5 mL of Buffered Peptone Water (BPW; Beijing Land Bridge Technology Co., Ltd., China) for pre-enrichment and incubated at 37 °C for 24 h. The enriched culture was subsequently transferred into Tryptic Soy Broth supplemented with 7.5% NaCl (TSB; Beijing Land Bridge Technology Co., Ltd., China) and incubated at 37 °C for an additional 16 h for selective enrichment, thereby improving the recovery efficiency of *S. aureus*. Following enrichment, one to two loopfuls of the culture were streaked onto Mannitol Salt Agar (MSA; Beijing Land Bridge Technology Co., Ltd., China) and incubated at 37 °C for 24 h. Presumptive *S. aureus* colonies were selected based on their colony morphology and purified by repeated streaking. Purified isolates were further inoculated onto a selective chromogenic medium specific for *S. aureus* (Guangdong Huankai Microbial Science and Technology Co., Ltd., China) and incubated at 37 °C for 24 h. Typical purple-red colonies were selected according to the manufacturer’s recommended chromogenic characteristics and cultured in Luria–Bertani broth for propagation. Presumptive *S. aureus* isolates were subsequently subjected to preliminary identification by amplification and sequencing of the 16S rRNA gene.

**Table 1:** Isolation of *S. aureus* from samples of different origins.

Specific origin	Number of strain	Number of samples	Separation rate (%)	Sours
Air/Dust	28	62	45.16%	Environmental Sample
Excreta/Filth	14	58	24.14%	Environmental Sample
Cage equipment	2	13	15.38%	Environmental Sample
Feed trough	19	198	9.60%	Environmental Sample
Surroundings/Water	3	300	1.00%	Environmental Sample
Digestive/Reproductive	2	11	18.18%	Clinical Sample
Internal organs	6	46	13.04%	Clinical Sample
Bone/Joint/Tendon	34	262	12.98%	Clinical Sample
Skin/Peripheral	7	54	12.96%	Clinical Sample
Embryo	11	440	2.50%	Clinical Sample

### 2.2 DNA extraction and WGS

Genomic DNA was extracted from the *S. aureus* isolates using a TIANamp Bacterial DNA Kit (Tiangen Biotech, Beijing, China) in accordance with the manufacturer’s instructions. DNA concentration and purity were assessed using a NanoDrop spectrophotometer (Thermo Fisher Scientific, USA), and DNA integrity was evaluated by electrophoresis on a 1% agarose gel. WGS was performed using the Illumina HiSeq platform (Illumina Inc., USA). Sequencing libraries were constructed to generate 150-bp paired-end reads, with an average sequencing depth of approximately 200 × per isolate. Raw paired-end reads were adapter-trimmed and quality-filtered with fastp v0.23.4, removing adapter sequences, low-quality bases (Phred < Q20), reads with >40% unqualified bases, and reads containing >5 ambiguous nucleotides (N) or shorter than 50 bp. The resulting high-quality reads were *de novo* assembled using Unicycler v0.5.0.^1^ The quality of the assembled genomes was assessed using CheckM v1.0.12 (Parks et al., 2015). Only high-quality genomes with an estimated completeness of >95% and contamination of <5% were retained for subsequent analyses.

### 2.3 Genomic characterization and sequence analysis

The genomes of the *S. aureus* isolates were functionally annotated using the Rapid Annotation using Subsystem Technology server (RAST)^2^ and Prokka v1.14.5. ABRicate^3^ was used to screen the genome assemblies against the ResFinder,^4^ Virulence Factor Database (VFDB),^5^ ISfinder,^6^ and PlasmidFinder^7^ databases to identify ARGs, virulence-associated genes, insertion sequences (ISs), and plasmid replicon types, respectively. To ensure the reliability of sequence-based identification, only matches with a nucleotide sequence identity of ≥90% and a query coverage of ≥90% were considered credible annotations. MLST was performed for all isolates using the *S. aureus* MLST scheme available in the PubMLST database.^8^ In parallel, *spa* typing was conducted using spaTyper^9^ based on polymorphisms within the repetitive region of the gene encoding staphylococcal protein A (*spa*).

### 2.4 Antimicrobial susceptibility testing by minimum inhibitory concentrations determination

MICs were determined by broth microdilution for 126 isolates. Resistance was analyzed for 11 antimicrobial agents, including penicillin G (PEN), ampicillin (AMP), amoxicillin (AMX), ceftiofur (TIO), cefquinome (CFQ), erythromycin (ERY), oxytetracycline (OTC), chlortetracycline (CTC), ciprofloxacin (CIP), sulfadiazine (SDZ), and sulfamonomethoxine (SMM). Resistance was assigned using agent-specific breakpoints from the Clinical and Laboratory Standards Institute (CLSI) M100.

### 2.5 Phylogenetic and pangenome analyses

Core-genome alignments were generated using Snippy v4.6.0^10^ (Seemann, 2014). The assemblies were mapped to the reference strain *S. aureus* NCTC 8325 (GenBank accession CP000253.1) using BWA-MEM, and SNPs were called using FreeBayes. Variants were filtered using the following criteria: minimum read depth of 10 × (−-mincov 10), minimum mapping quality of 60 (−-min-mapping-quality 60), minimum variant quality of 100 (−-minqual 100), and minimum alternate-allele fraction of 0.9 (−-minfrac 0.9). High-quality core-genome SNP sites present across all isolates were then extracted using snippy-core for subsequent phylogenetic reconstruction and transmission analysis. A maximum-likelihood phylogenetic tree was reconstructed from the core-genome SNP alignment using IQ-TREE 2 (Minh et al., 2020). The best-fitting nucleotide substitution model was automatically selected using ModelFinder. The resulting phylogenetic tree was visualized and annotated using the Interactive Tree of Life web server (iTOL).^11^ Metadata used for tree annotation included sample source, molecular typing information, and the presence or absence of antimicrobial resistance and virulence genes. The population structure of the *S. aureus* isolates was assessed using RhierBAPS to identify potential genetic populations and hierarchical genomic clusters (Tonkin-Hill et al., 2018).

### 2.6 Statistical analysis

All statistical analyses were performed using R software version 4.4.3. Differences in the detection rates of *S. aureus* among different sample types were evaluated using Pearson’s chi-square test or Fisher’s exact test, as appropriate. Differences in the prevalence of ARGs among bacterial populations were assessed using the chi-square test. Correlations among ARGs, insertion sequences, and plasmid replicons were evaluated using correlation analysis implemented with the corrplot package in R. All statistical tests were two-sided, and a *p* value of <0.05 was considered statistically significant.

### 2.7 Animals and ethics statement

All animal care was conducted under strict supervision by the Animal Management of Sichuan Agricultural University. The experiment involving chicken was approved by the Animal Ethics Committee (AEC) of the Sichuan Agricultural University (License: SACU-1428). Briefly, all the birds were sacrificed for procuring the samples using carbon dioxide, followed by exsanguination. At the end of the experiments, the birds were intravenously injected with pentobarbital sodium.

## 3 Results

### 3.1 ST398 lineage of *Staphylococcus aureus* exhibits widespread dissemination in chicken farms

A total of 1,444 samples were collected from 56 chicken farms across eight regions of Sichuan Province, China, yielding 126 *S. aureus* isolates, with an overall isolation rate of 8.73% (126/1444; Figure 1A). Based on the sample origin, all isolates were classified into two groups: clinical isolates (*n* = 60) and environmental isolates (*n* = 66). Comparison of *S. aureus* isolation rates across different sample types showed that digestive/reproductive samples had the highest positivity rate among clinical samples (2/11, 18.18%), followed by internal organ samples (6/46, 13.04%; Figure 1B). Among environmental samples, *S. aureus* was most frequently detected in air/dust samples (28/62, 45.16%), followed by excreta/filth samples (14/58, 24.14%). We subsequently analyzed the source distribution of the 126 isolates and found that clinical isolates were mainly recovered from bone/joint/tendon tissues (*n* = 32) and embryo samples (*n* = 11), whereas environmental isolates were predominantly obtained from air/dust samples (*n* = 27) and feed troughs (*n* = 19), indicating a broad distribution of *S. aureus* within the chicken production environment (Figure 1C). WGS followed by *spa* typing and MLST, was performed for all 126 *S. aureus* isolates (Figure 1D). *spa* typing revealed that the isolates were mainly distributed among t2582, t002, t12359, t2247, t001, and several other minor *spa* types. Among these, t2582 and t002 were highly prevalent across isolates from different sources, suggesting that these clonal lineages may possess enhanced environmental adaptability and host colonization potential. MLST analysis further demonstrated that ST398 was the predominant sequence type among poultry-associated *S. aureus* isolates in this region, followed by ST5, ST692, and several unassigned sequence types (Unknown). Collectively, these findings indicate that ST398 *S. aureus* has emerged as the dominant circulating lineage in chicken production systems in Sichuan Province and may undergo persistent transmission and dissemination within farm environments.

**Figure 1:**
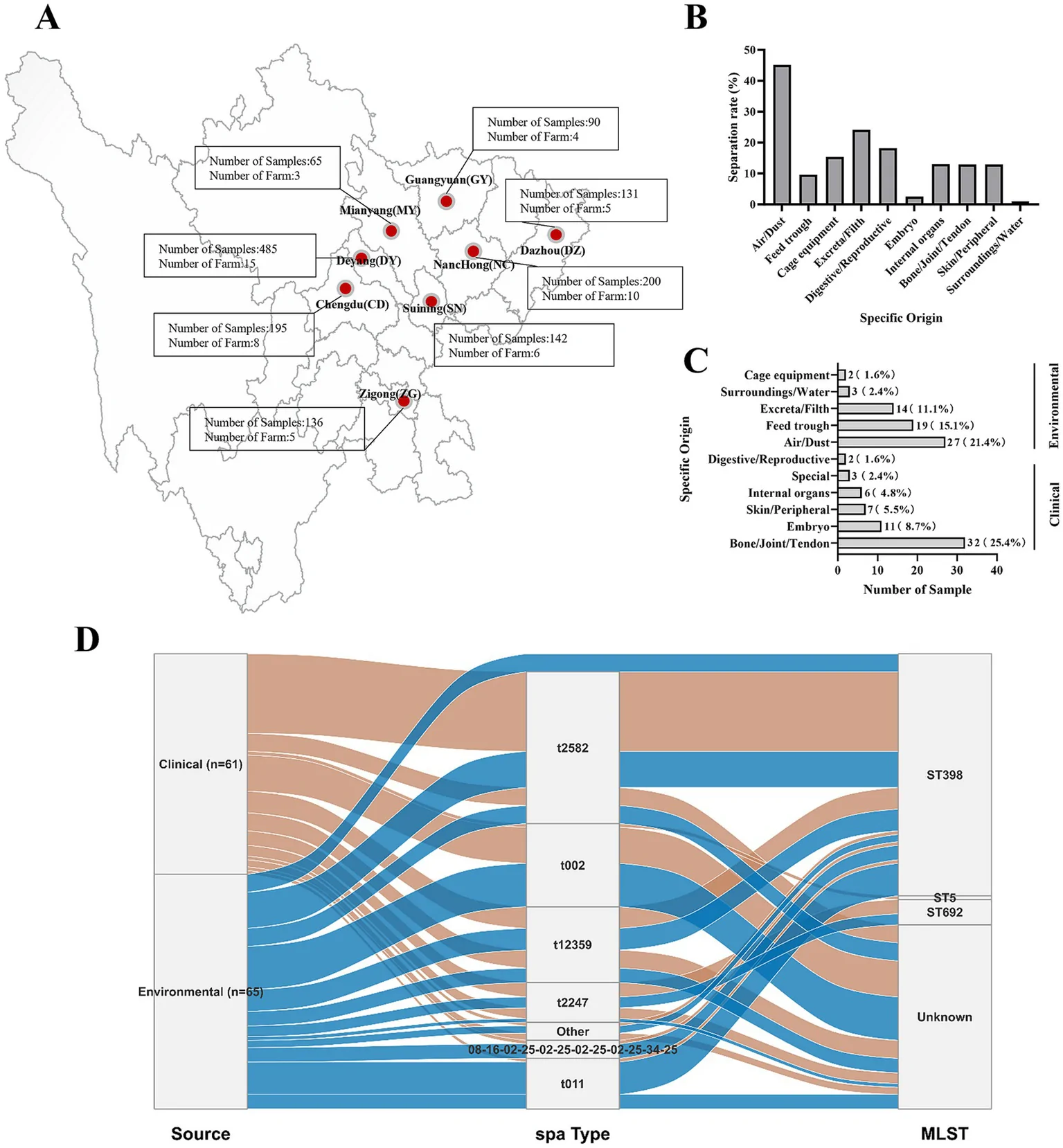
Isolation, source distribution, and molecular typing of *S. aureus* isolates. **(A)** Map showing the geographical distribution of sampling sites. **(B)** Isolation rates of *S. aureus* from different sample types and representative colony morphology on selective agar. **(C)** Distribution of the sampling sources of the 126 *S. aureus* isolates. **(D)** Sankey diagram showing the relationships among isolate sources, *spa* types, and MLST profiles. Samples were classified into two major categories according to their source: “Clinical Sample” (brown) and “Environmental Sample” (blue). The middle column represents the *spa* types, while the rightmost column shows the MLST profiles of the 126 isolates.

### 3.2 Poultry-associated *Staphylococcus aureus* isolates harbor diverse ARGs and virulence determinants

To investigate the differences in antimicrobial resistance profiles and pathogenic potential between clinical and environmental *S. aureus* isolates, ARGs and virulence-associated genes were systematically characterized based on WGS data. A total of 35 distinct ARGs were identified among the 126 isolates. The most frequently detected ARGs included *blaZ* (122/126, 96.83%), *tet(L)* (116/126, 92.06%), *dfrE* (103/126, 81.74%), *fexA* (89/126, 70.63%), *aac(6′)-aph(2″)* (70/126, 55.56%), *ant(6)-Ia* (70/126, 55.56%), *lnu(B)* (70/126, 55.56%), and *lsa(E)* (70/126, 55.56%). Notably, the key methicillin resistance determinant *mecA*, which is associated with MRSA, was detected in only five isolates, accounting for 3.97% of all recovered strains (Figure 2A; Supplementary Table 1). Comparative analysis of ARG profiles between isolates from different sources revealed a distinct enrichment pattern of several resistance determinants in environmental isolates. Specifically, *aadD*, *lnu(B)*, *lsa(E)*, *aac(6′)-aph(2″)*, *ant(6)-Ia*, *erm(C)*, and *tet(K)* were predominantly identified in environmental isolates, whereas their prevalence was relatively lower among clinical isolates. These findings suggest that environmental niches within chicken farms may serve as important reservoirs for antimicrobial resistance determinants. To further explore the potential genetic linkage among resistance-associated elements, a correlation network analysis was constructed based on ARGs, ISs, and plasmid replicons (Figure 2B). Multiple significant associations were observed among these genetic components. For example, strong correlations were identified between *blaZ* and *tet(L)*, as well as between the plasmid replicon repUS43 and *dfrG*, suggesting that these resistance determinants may undergo co-selection and coordinated dissemination through shared genetic backgrounds or mobile genetic elements. The antibiotic usage profiles of the four source farms were analyzed, revealing that Farm1 exhibited the highest usage of doxycycline hydrochloride and florfenicol. Notably, 30% of isolates from Farm1 carried doxycycline hydrochloride-related resistance genes, while 45% carried florfenicol-related resistance genes, indicating that prolonged antibiotic selection pressure has driven the enrichment and clonal expansion of resistant strains in this environment (Supplementary Table 2). Subsequently, we determined the major antimicrobial resistance phenotypes of these isolates. The MIC results showed that the 126 *Staphylococcus aureus* isolates showed marked differences in resistance frequencies across the 11 antimicrobial agents. Resistance was most frequent to penicillin G and ampicillin (both 100%, 126/126), followed by amoxicillin (99.2%, 125/126), ciprofloxacin (98.4%, 124/126), oxytetracycline (96.8%, 122/126), erythromycin (94.4%, 119/126), chlortetracycline (88.1%, 111/126), and sulfadiazine (80.2%, 101/126). Lower resistance frequencies were observed for sulfamonomethoxine (7.1%, 9/126), ceftiofur (3.2%, 4/126), and cefquinome (1.6%, 2/126; Figure 1A). When these agents were grouped into five broad antimicrobial classes, 95 isolates (75.4%) met the resistance thresholds for at least one agent in all five classes, while 28 (22.2%) and three (2.4%) did so in four and three classes, respectively (Figure 1B). This class-level analysis was restricted to the 11 agents with resistance thresholds recorded in the dataset Supplementary Figures 1A,B).

**Figure 2:**
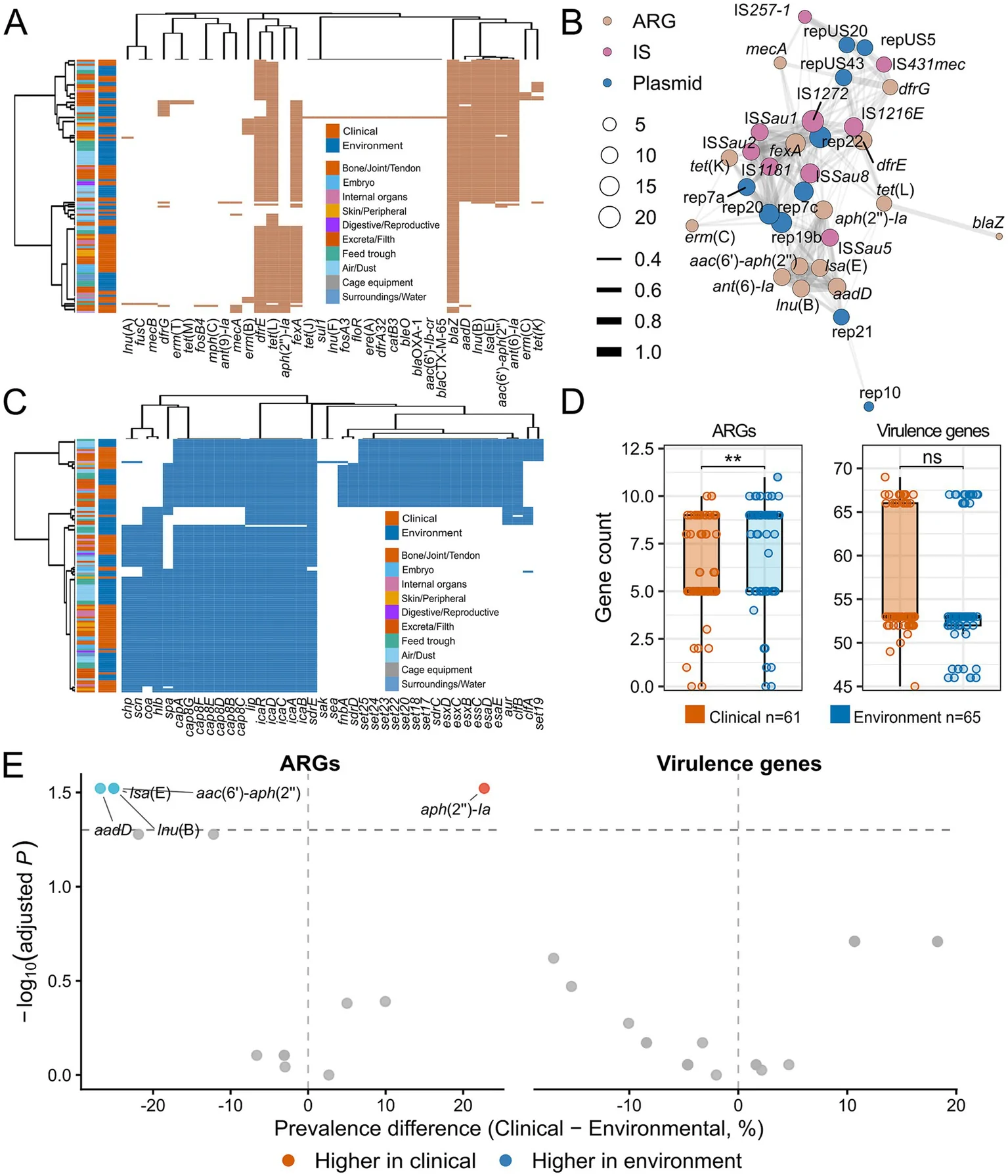
Distribution of antimicrobial resistance and virulence genes among the 126 *S. aureus* isolates. **(A)** Clustered heatmap showing the distribution of ARGs among the 126 *S. aureus* isolates. **(B)** Correlation network among ARGs, insertion sequences, and plasmid replicons. **(C)** Clustered heatmap showing the distribution of virulence-associated genes among all isolates. **(D)** Comparison of the numbers of ARGs and virulence-associated genes carried by clinical and environmental isolates. **(E)** Volcano plot showing ARGs and virulence-associated genes that differed significantly in prevalence between clinical and environmental isolates.

A total of 41 virulence-associated genes were identified among the investigated isolates. The most prevalent virulence determinants included adhesion-related genes associated with biofilm formation, such as *sdrE*, *icaA*, *icaB*, *icaC*, *icaD*, and *icaR;* core genes involved in capsular polysaccharide biosynthesis, including *capA* and *cap8A-G*; and enzyme-associated virulence factors, including *coa*, *hlb*, and *lip* (Figure 2C). In addition, genes involved in bacterial adhesion and colonization (*fnbA*, *sdrC*, *sdrD*, *clfA*, and *clfB*), enterotoxin-associated genes (*set17*, *set18*, *set20*, *set23*, *set24*, and *set25*), and immune evasion-associated genes (*esxB*, *esxC*, *esxD*, *essC*, *esaD*, and *esaE*) were detected only in a subset of isolates. Comparison of the per-isolate gene carriage between clinical (*n* = 61) and environmental (*n* = 65) isolates demonstrated that environmental isolates carried significantly more ARGs than clinical isolates (mean 7.40 ± 2.78 vs. 6.25 ± 2.53; median 9 vs. 5; Mann–Whitney U test, *p* = 0.005). In contrast, the number of virulence-associated genes did not differ significantly between the two groups (mean 55.25 ± 6.97 vs. 56.57 ± 6.70; median 53 vs. 53; *p* = 0.250; Figure 2D). Further statistical analysis identified significant differences in the distribution of several resistance genes between environmental and clinical isolates. After Benjamini-Hochberg FDR correction for multiple comparisons, four ARGs remained significantly more frequent in environmental isolates (*aadD*, *aac*(6′)-*aph*(2″), *lnu*(B), and *lsa*(E); q = 0.030), and *aph*(2″)-*Ia* was significantly more frequent in clinical isolates (q = 0.030; Figure 2E; Supplementary Figure 1C).

### 3.3 Poultry-associated *Staphylococcus aureus* exhibits substantial genetic diversity and cross-source clustering patterns

To elucidate the genetic relatedness and evolutionary relationships among the 126 poultry-associated *S. aureus* isolates, a maximum-likelihood phylogenetic tree was constructed based on core-genome SNPs. The phylogenetic framework was further integrated with information regarding isolate origin, MLST profiles, *spa* types, plasmid replicons, ARG profiles, and virulence-associated gene repertoires for comprehensive comparative analysis (Figure 3). Phylogenetic analysis revealed that the 126 *S. aureus* isolates were primarily clustered into two major genetic lineages. The first lineage was mainly composed of t002- and t2247-type isolates, which exhibited a relatively higher abundance of virulence-associated genes, indicating a potential enrichment of pathogenicity-related traits within this lineage. The second lineage contained diverse *spa* types, including t011, t034, t2876, and several other minor types, displaying greater genetic heterogeneity. Notably, isolates from both clinical and environmental sources were simultaneously distributed within the two major phylogenetic clusters, suggesting that strains originating from different ecological niches shared closely related genetic backgrounds. However, several subsets of clinical and environmental isolates exhibited clear clustering patterns, indicating potential epidemiological connections between farm environments and infected hosts.

**Figure 3:**
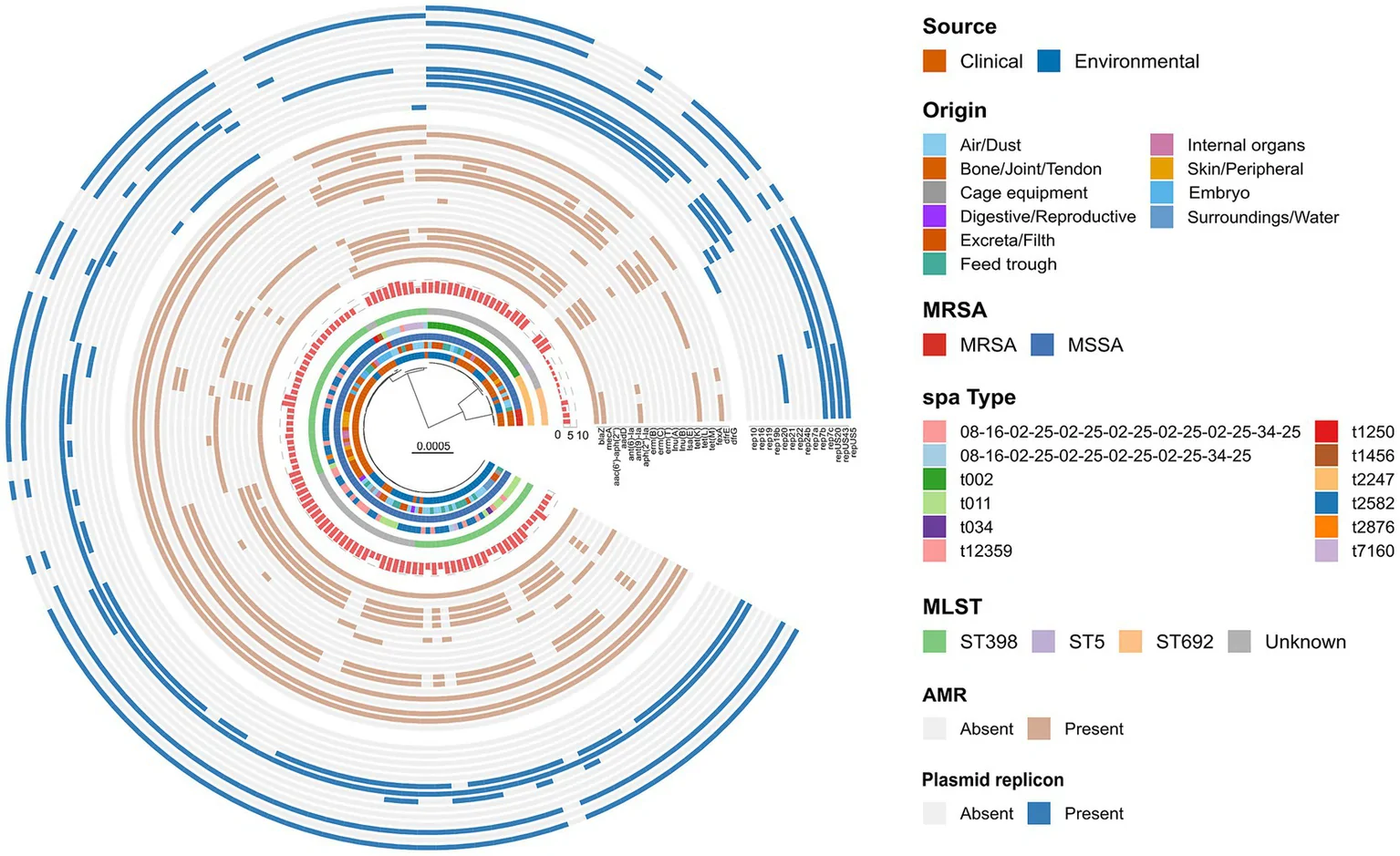
Maximum-likelihood phylogenetic tree of the 126 *S. aureus* isolates. The annotation rings, from the outermost to the innermost, represent plasmid replicons (blue, present; white, absent), ARG(brown, present; white, absent), the number of ARGs carried by each isolate (red bars), MLST profiles (color-coded), *spa* types (color-coded), methicillin-resistance status (red, MRSA; blue, MSSA), specific isolation sources (color-coded), and source categories (orange, clinical samples; blue, environmental samples).

### 3.4 SNP-based genomic analysis of *Staphylococcus aureus* revealed the genetic relatedness among isolates from different sources

To further assess the potential transmission risk of *S. aureus* within chicken production systems, genetic relatedness among isolates was assessed based on pairwise core-genome SNP distances (Supplementary Table 3). The SNP-based phylogenomic analysis showed a high degree of concordance with the *spa* typing results, with all isolates generally grouped into two major genetic populations (Figure 4A). A large number of closely related isolate pairs were identified between clinical and environmental sources, indicating genetic exchange and circulation of *S. aureus* across different ecological compartments within chicken farms (Figure 4B). These findings indicate that poultry-associated *S. aureus* dissemination is not restricted to a single reservoir but involves clonal expansion and potential cross-source dissemination throughout the farm ecosystem. Strengthening biosecurity measures, improving environmental hygiene, and reducing contamination of critical farm facilities may represent effective strategies for limiting the persistence and dissemination of *S. aureus* in poultry production systems.

**Figure 4:**
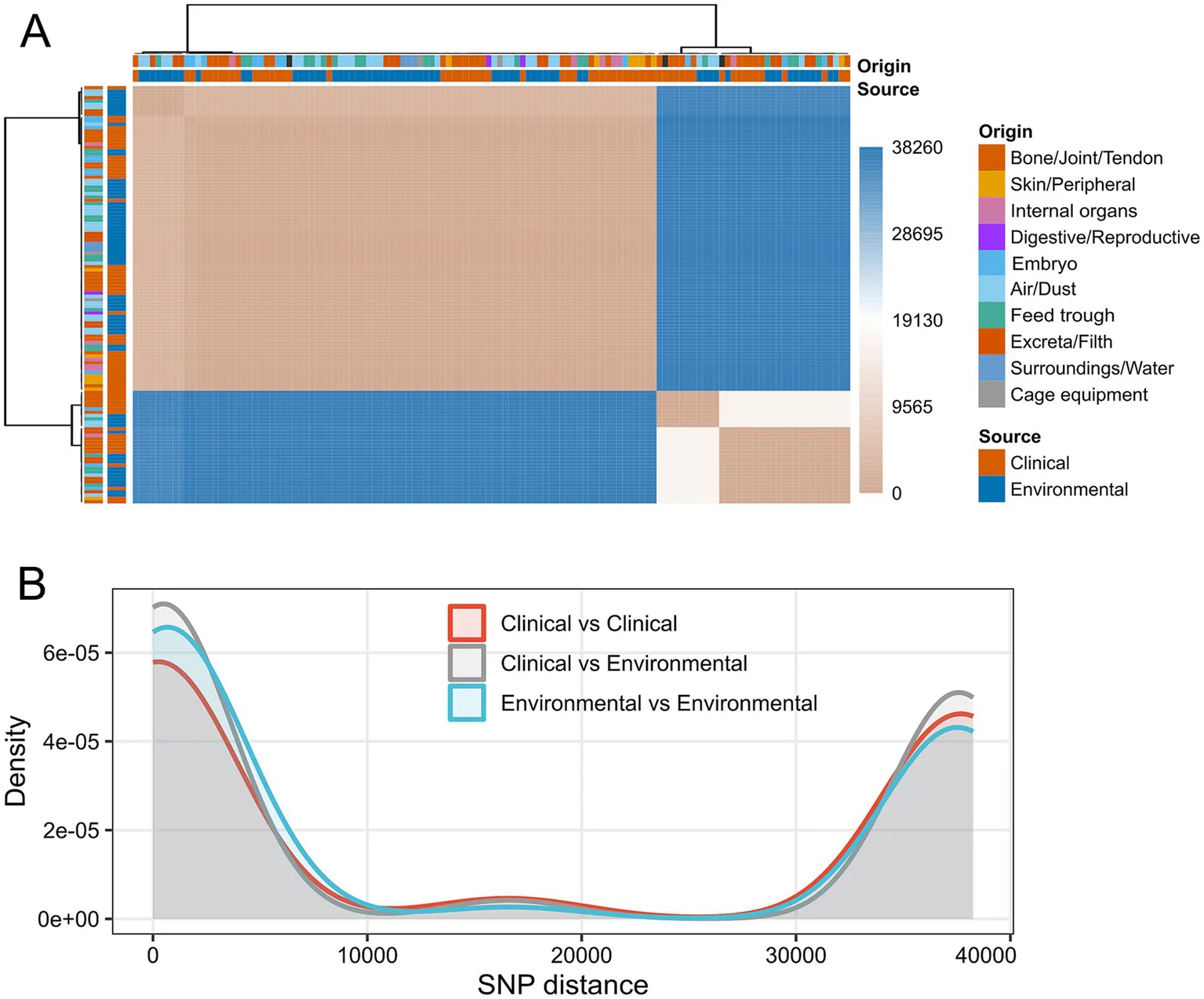
Core-genome SNP-based analysis of potential transmission among the 126 *S. aureus* isolates. **(A)** Heatmap showing pairwise core-genome SNP distances among isolates from different sources. **(B)** Kernel density distributions of pairwise core-genome SNP distances among the 126 *S. aureus* isolates.

## 4 Discussion

In recent years, the continued intensification of livestock and poultry production, together with increasing antimicrobial selection pressure, has led animal-associated *S. aureus* to emerge as an important interface linking animal health, food safety, and public health (Fang et al., 2024). In this study, whole-genome analysis of 126 poultry-associated *S. aureus* isolates revealed the predominance of the ST398/t2582 clone in chicken production systems in Sichuan Province. Numerous studies have demonstrated that ST398 has become one of the most successful animal-associated *S. aureus* lineages worldwide (Huang and Chen, 2020; Lei et al., 2025). Compared with traditionally recognized human-associated ST398 populations, livestock-associated ST398 generally exhibits enhanced environmental adaptability and can withstand prolonged antimicrobial selection pressure through the acquisition of resistance-associated mobile genetic elements (Cui et al., 2025; Hu et al., 2025). In this study, ST398 was identified as the predominant MLST type among poultry-associated *S. aureus* isolates in Sichuan Province, whereas *spa* typing further identified t2582 as the major circulating type. Notably, previous investigations of ST398 have mainly focused on swine production systems (Kawanishi et al., 2024; Lee et al., 2024; Zou et al., 2022), whereas our findings demonstrate that ST398/t2582 also exhibits a clear epidemiological advantage in poultry production environments, suggesting that this clone may possess a broader host range and greater ecological adaptability than previously recognized.

The occurrence of antimicrobial-resistant bacteria in poultry populations represents a substantial threat to human health. MRSA, which is primarily associated with carriage of the *mecA* gene, remains a major public health concern, with an estimated 130,000 deaths globally attributable to MRSA infections in 2021 (Parsons et al., 2026). In the present study, MRSA was detected in both clinical and environmental samples from chicken farms. Notably, the five MRSA isolates originated from different chicken farms. Although their isolation rate was low, the risk of MRSA transmission within chicken farms warrants further investigation. Nevertheless, more than 90% of the isolates carried *blaZ* and *tet(L)*, indicating that poultry production systems may act as reservoirs for multidrug resistance determinants. Importantly, environmental isolates carried significantly more ARGs than clinical isolates, and several aminoglycoside-, macrolide-, and lincosamide-resistance genes were predominantly enriched among environmental isolates. These findings indicate that the farm environment may represent a critical reservoir for multidrug-resistant strains and an important ecological compartment for horizontal gene exchange. The pronounced enrichment of doxycycline hydrochloride and florfenicol-related resistance genes in Farm1 underscores the direct impact of selective antibiotic pressure on the genomic landscape of commensal and pathogenic bacteria in intensive poultry systems. These findings align with global evidence that sustained use of critically important antimicrobials accelerates the fixation of resistance determinants within bacterial populations, necessitating proactive resistance management (Li et al., 2025; Shen et al., 2023). The frequent resistance observed across several antimicrobial classes indicates a substantial resistance burden within this collection of *S. aureus*. The contrasting results for penicillins and cephalosporins, and for the two sulfonamides, highlight variation among agents within broad antimicrobial groups. Although all isolates met resistance thresholds in at least three of the five evaluated classes, this represents an operational classification based on the selected agents rather than a comprehensive assessment of multidrug resistance. To mitigate the long-term risk of cross-farm transmission and therapeutic failure, we advocate for the implementation of a structured antimicrobial stewardship program: ‌regular bacterial isolation and phenotypic susceptibility profiling‌ should be established as routine surveillance, forming a ‌longitudinal antimicrobial resistance database‌ specific to regional poultry production.

In addition to antimicrobial resistance, the persistent circulation of *S. aureus* is closely associated with its ability to survive in the environment and adapt to animal hosts (Uhlemann et al., 2017; Uhlemann et al., 2012). A total of 41 virulence-associated genes were identified in this study. Virulence determinants involved in biofilm formation, adhesion, colonization, and extracellular matrix binding were widely distributed among the isolates. Previous studies have suggested that the successful dissemination of animal-associated *S. aureus* does not necessarily depend on highly invasive virulence phenotypes, but may instead be driven by traits that promote long-term survival, persistence, and ecological adaptation (Nakatsuji et al., 2023; Silva et al., 2023). Biofilm formation facilitates bacterial attachment to farm facilities, drinking-water equipment, and animal tissues. It can also enhance bacterial survival under adverse conditions by reducing antimicrobial penetration and increasing tolerance to oxidative stress (Choi and Kim, 2024; Do et al., 2025; Potapova et al., 2025). In the present study, no significant difference was observed in the total number of virulence-associated genes carried by environmental and clinical isolates. This finding suggests that environmental *S. aureus* populations should not simply be regarded as low-virulence contaminants, as they may retain substantial colonization and infection potential.

WGS-based phylogenetic analysis has become an essential approach for elucidating the epidemiology of animal-associated pathogens. Compared with conventional typing methods, such as MLST and *spa* typing, core-genome SNP analysis provides substantially higher resolution for determining genetic relatedness and identifying recent transmission events (Duchez et al., 2025; Seah et al., 2023). In the present study, the core-genome SNP-based phylogeny showed that clinical and environmental isolates were interspersed across multiple phylogenetic lineages rather than forming completely distinct populations. This pattern indicates close genetic relatedness and ongoing epidemiological connectivity between isolates associated with clinical infections and those recovered from the farm environment. These findings provide further evidence of potential cross-source transmission of *S. aureus* within poultry production systems. In particular, air and dust may serve as potential transmission routes within poultry houses. Recent studies have detected MRSA and multidrug-resistant *Staphylococcus* spp. in poultry-house bioaerosols and downwind air, while indoor-outdoor comparisons have revealed overlapping bacterial communities and antimicrobial resistance profiles, supporting the potential release and dispersal of poultry-associated bacteria from confinement buildings into surrounding environments. Compared with direct-contact transmission, aerosol-mediated spread is less visible and may occur continuously over extended periods, potentially explaining the broad distribution of environmental isolates observed in this study. Furthermore, feed troughs and drinking-water systems are repeatedly exposed to animal contact and fecal contamination and may therefore provide favorable sites for bacterial colonization, persistence, and subsequent dissemination.

## 5 Conclusion

This study systematically characterized the genomic epidemiology of *S. aureus* in poultry production systems in Sichuan Province and identified ST398/t2582 as the predominant circulating clone. Our findings also highlight the central role of the farm environment in maintaining antimicrobial resistance determinants and facilitating the dissemination of *S. aureus* across different ecological compartments. These results expand current understanding of the ecological adaptation of poultry-associated *S. aureus* and emphasize the importance of continuous surveillance and integrated control of animal-associated antimicrobial-resistant bacteria under a One Health framework. Effective intervention strategies should therefore combine routine genomic surveillance, improved environmental sanitation, enhanced air-quality management, and targeted disinfection of drinking-water and feeding systems to reduce the persistence and transmission of *S. aureus* within chicken farms.

## Data Availability

The datasets presented in this study can be found in online repositories. The names of the repository/repositories and accession number(s) can be found at: https://www.ncbi.nlm.nih.gov/genbank/, PRJNA1498777.

## Footnotes

1
https://github.com/rrwick/Unicycler

2
https://rast.nmpdr.org/

3
https://github.com/tseemann/abricate

4
https://github.com/cadms/resfinder

5
http://www.mgc.ac.cn/VFs/

6
https://isfinder.biotoul.fr/about.php

7
https://github.com/kcri-tz/plasmidfinder

8
https://pubmlst.org/

9
https://github.com/HCGB-IGTP/spaTyper

10
https://github.com/tseemann/snippy

11
https://itol.embl.de/

## Supplementary material

SUPPLEMENTARY FIGURE 1 **(A)** MIC analysis of 126 *S. aureus* isolates against 11 major antibiotics. **(B)** Number of multidrug-resistant isolates. **(C)** Heatmap of ARGs and virulence gene distribution carried by 126 *S. aureus* isolates.

SUPPLEMENTARY TABLE 1 Background information and genomic characteristics of the 126 *S. aureus* isolates.

SUPPLEMENTARY TABLE 2 Antibiotic usage profiles and ARG carriage profiles of four farms.

SUPPLEMENTARY TABLE 3 The total assembly length, number of contigs, N50, and L50 for all 126 genomes.
